# Young generations’ hopelessness perpetuates long-term conflicts

**DOI:** 10.1038/s41598-023-31667-9

**Published:** 2023-03-25

**Authors:** Béatrice S. Hasler, Oded A. Leshem, Yossi Hasson, Daniel H. Landau, Yara Krayem, Chen Blatansky, Guy Baratz, Doron Friedman, Charis Psaltis, Huseyin Cakal, Smadar Cohen-Chen, Eran Halperin

**Affiliations:** 1 Sammy Ofer School of Communications, Reichman University, Herzliya, Israel; 2 Department of Psychology, The Hebrew University of Jerusalem, Jerusalem, Israel; 3 Department of Media, School of Arts, Design and Architecture, Aalto University, Espoo, Finland; 4 Baruch Ivcher School of Psychology, Reichman University, Herzliya, Israel; 5 Department of Psychology, University of Cyprus, Nicosia, Cyprus; 6 School of Psychology, Keele University, Keele, UK; 7 Sussex Business School, University of Sussex, Sussex, UK; 8 Sagol School of Neuroscience, Tel Aviv University, Tel Aviv, Israel

**Keywords:** Psychology, Human behaviour

## Abstract

Transforming long-term conflicts into peaceful intergroup relations is one of the most difficult challenges for humanity. Such meaningful social changes are often driven by young people. But do young people living in contexts of long-term conflicts believe that change is even possible? In a series of six studies (*N*_*total*_ = 119,671) over two decades and across two unrelated intractable conflicts in Israel/Palestine and Cyprus, we found that younger (compared to older) generations from both respective rival groups have less hope for peace, and consequently less conciliatory attitudes. We also show that this gradual improvement of peace-promoting emotions and attitudes with increasing age can be experimentally accelerated in young people through a virtual reality-based aging simulation. These findings provide a new perspective on the fundamental question of why long-term conflicts are so difficult to resolve and highlight the importance of instilling hope in young generations to advance peace processes.

## Introduction

Intractable conflicts between national, ethnic, or religious groups are one of the world’s greatest challenges. Their peaceful resolution requires difficult and sometimes risky decisions and actions. One of the main catalysts of peace-supporting attitudes and actions is hope^1,2^, which has been found to reduce the desire to retaliate and increase willingness to forgive^3^, to make concessions and compromise for peace^4^, and to provide humanitarian aid to the adversary^5^.

Hope is a mental state elicited when one desires a goal and has some belief (though not certainty) in its fulfillment^6,7^. Hope is thus comprised of two dimensions, a motivational dimension linked to desires (i.e., wishes or aspirations) for a goal and a cognitive dimension linked to the assessments of the probability that the goal will be attained^8–10^. The higher the desires and beliefs in the possibility of attainment, the higher the hope. In conflicts that are characterized by repeatedly failed peace negotiations, however, hope deteriorates as the years go by^1^. In such contexts, hopelessness becomes a long-term sentiment that leads to apathy and unwillingness to create change, and thereby contributes to the perpetuation and continuation of the conflict^11,12^. While it seems reasonable that individuals involved in long-term conflicts still desire the end of the conflict, their beliefs in the possibility of a peaceful resolution of the conflict are typically low^8^.

Young people play a particularly crucial role in promoting meaningful social change as evidenced by many youth-driven movements and revolutions around the world throughout history^13,14^. Yet, young people’s motivation and engagement in peace-building processes largely depend on whether they believe that peace is possible. If young generations of societies involved in intractable conflicts lack hope, this would dramatically reduce the chances for a peaceful resolution of such conflicts. However, it is unclear whether and how age is related to hope for peace and conciliatory attitudes.

Hope requires the ability to imagine the future, and involves higher mental processes, including imagery, cognitive flexibility, and mental exploration of novel situations^15,16^. Accumulating empirical evidence suggests that the age-related decline in episodic memory of past events extends to an impairment in the ability to imagine future events^17^ as both processes rely on the same neural mechanisms^18^. Research has found that people do not only rely on memories of past events when anticipating their personal future but also when imagining their nation’s collective future^19^. This might also be relevant in a conflict context, where older people have seen many futile attempts of negotiations and lived under the conditions of conflict for a much longer time than younger people. These factors may result in a reduced ability of older individuals to imagine an alternative, peaceful future, which may be associated with lower levels of hope for peace. Furthermore, previous research has found that openness decreases with age^20^ and that older generations typically hold more conservative attitudes^21^, are less tolerant^22^ and more prejudiced^23^ concerning sociopolitical issues than their younger counterparts. Contrary to the intuitive assumption derived from these previous findings—that hope for peace and conciliatory attitudes decrease with age—we argue that the opposite might be the case as indicated by related previous research in conflict contexts.

The hypothesis that young people involved in intractable conflicts lack hope is plausible because young people may have a more static perception of the conflict. Static perceptions have been found to be negatively related to hope for peace and conciliatory attitudes^24,25^. Older people, on the other hand, have a broader perspective as they have experienced fluctuations and changes of the conflict throughout their lifespan, which may be associated with a stronger belief that peace is possible and consequently more conciliatory attitudes. Some initial indications supporting this proposition can be found in research showing that young people lack the wisdom of older adults that is associated with better conflict resolution skills^26^.

Our hypothesis of the young generations’ hopelessness is further supported by previous research that has found a general tendency in youth to pay more attention to and remember more negative than positive events, which is assumed to be grounded in evolutionary survival advantages^27^. This negativity bias in youth has been found to decrease with age^28^. According to socio-emotional selectivity theory of aging^29^, the perception of limited remaining time, that is naturally associated with aging, motivates older people to regulate their emotions and shift their goals towards more positive and away from negative experiences. Numerous studies across different cultures^30–32^ have demonstrated the age-related positivity (or reduced negativity) effect in memory, attention, and information processing (see^28^ for a review). The effect has also been found to extend to future events that were rated more positively by older compared to younger adults^33^. According to the appraisal-based framework of emotions^15^, applied to hope specifically^5,12^, age-related changes in the appraisal of conflict events (towards reduced negativity in how events are remembered, and what type of conflict-related information is attended to and how it is processed) may influence hope sentiments, and thereby result in more conciliatory attitudes with increasing age.

We tested the hypothesized generational gap in hope for peace and resulting conciliatory attitudes in a series of six studies in the contexts of the Israeli–Palestinian conflict and the conflict in Cyprus—two prolonged ethnonational conflicts that have been ongoing for more than 60 years, and that have witnessed numerous unfruitful attempts of resolution.

## Results

### Study 1

In a first exploratory study, we analyzed existing data of monthly public opinion surveys conducted by the Israel Democracy Institute between 1994 and 2017 among Israeli Jews (*N* = 117,131) about the perceived probability of peace in the Israeli-Palestinian conflict. In 245 out of the 250 surveys we identified a positive correlation between age and subjective ratings of the probability of peace (index ranging from 0 = low probability to 1 = high probability).

**Figure 1:**
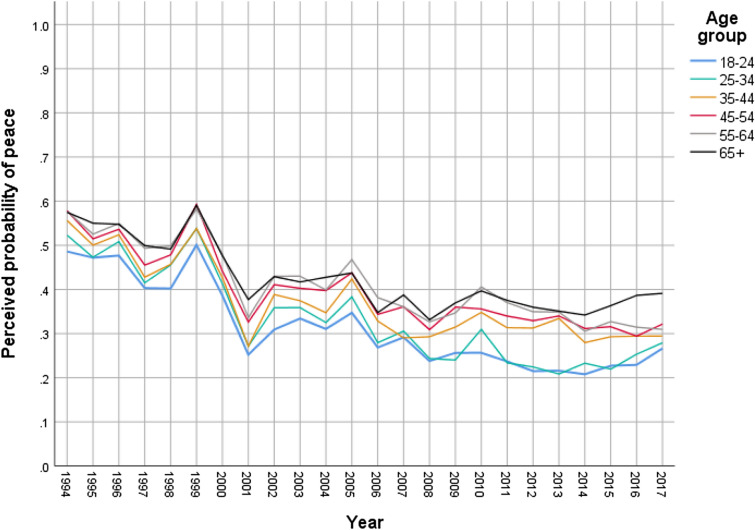
Yearly means of perceived probability of peace across age groups in surveys on Israeli Jews from 1994 to 2017 (1 indicates the highest level). *Data courtesy of the Guttman Center for Public Opinion and Policy Research at the Israel Democracy Institute. *Age groups and yearly means of perceived probability of peace are used for visualization of the linear trend. Analyzes are conducted using monthly ratings of perceived probability of peace and the continuous age variable.

A meta-analysis of the 250 surveys indicated a significant overall effect: mean *r* = 0.12, *SE* of mean *r*_*z*_ = 0.003, *Z* (Mean *r*_*z*_/*SE*) = 39.73, *p* < 0.001, *r*_*z*_ 95% CI 0.11–0.12. This finding reflects a consistent pattern of younger generations’ greater hopelessness regarding the resolution of the conflict compared to older generations (see Fig. 1). This is irrespective of time and context-dependent variations in the levels of hope across all age groups; as evident by generally higher levels of hope during the Oslo peace process (1993–1995) and a steep drop in a phase of violent escalation and cessation of the peace process during the second Palestinian uprising (Intifada) starting in 2000. Although the levels of hope generally decreased over time, *β* = − 0.20, *SE* < 0.001, *p* < 0.001, the gap between the younger and older generations significantly increased as indicated by the increasing strength of the age-hope correlation over time, *β* = 0.38, *SE* < 0.001, *p* < 0.001. A significant interaction between age and time on perceived probability of peace indicates a stronger decline in hope among the younger generations, *β* = − 0.11, *SE* < 0.001, *p* < 0.001. Thus, the context of no negotiation and no opportunities for peace appears to have stronger effects on the younger compared to the older generations.

If younger people are consistently less hopeful about the resolution of the conflict than older people, the question arises whether this effect is specific to hope related to the conflict; or alternatively, whether our findings indicate a broader phenomenon related to the correlation between age and dispositional optimism or general hope about the future.

### Study 2

Study 2 examined this question among Israeli Jews (*N* = 209) using validated measures of hope for peace^25^, general hope about the future^34^, and dispositional optimism^35^. All variables were measured using six-point scales, with six indicating the highest level. As the variables are not normally distributed, we used a non-parametric statistical test (Wilcoxon Signed-Rank Test) for median comparisons. Typical to the context of intractable conflict, hope for peace (*M*_*d*_ = 3.80) was rated significantly lower than both general hope about the future (*M*_*d*_ = 4.00), *W* = − 4.68, *p* < 0.001, and dispositional optimism (*M*_*d*_ = 4.50), *W* = − 5.61, *p* < 0.001. Hope for peace was positively correlated with general hope about the future, *ρ* = 0.40, *p* < 0.001, but not with dispositional optimism, *ρ* = 0.05, *p* = 0.47.

Consistent with the findings of Study 1, age significantly predicted hope for peace, *β* = 0.16, *SE* = 0.01, *p* = 0.02 (*β* = 0.16, *SE* = 0.01, *p* = 0.02, when controlling for individual variances in dispositional optimism). But age neither predicted general hope about the future, *β* = − 0.05, *SE* = 0.01, *p* = 0.47, nor dispositional optimism, *β* = 0.01, *SE* = 0.01, *p* = 0.92. If age is specifically related to hope for peace, does it also translate to more conciliatory attitudes among older compared to younger people, and can these age differences be found on both sides of the Israeli–Palestinian conflict?

### Study 3

Study 3 examined the relationship between age, hope for peace, and conciliatory attitudes (measured by ratings of support for peace-promoting policies) among Palestinian citizens of Israel (*N* = 174; Study 3a) and Jewish-Israelis (*N* = 984; Study 3b). Conciliatory attitudes were measured on six-point scales in both studies, with 6 indicating the highest level. Study 3a used an aggregated hope for peace measure ranging from 0 (low) to 100 (high). Study 3b measured hope for peace on a six-point scale (6 = highest level).

Since dovish, left-wing political ideology has been found to be associated with greater hope for peace^1,2,36–38^ and willingness to compromise for peace^4,37^, we controlled for political ideology in Study 3b. Political ideology was not measured in Study 3a since the right/left-wing categorization cannot be easily determined in the Palestinian minority group within the Jewish state of Israel.

**Figure 2:**
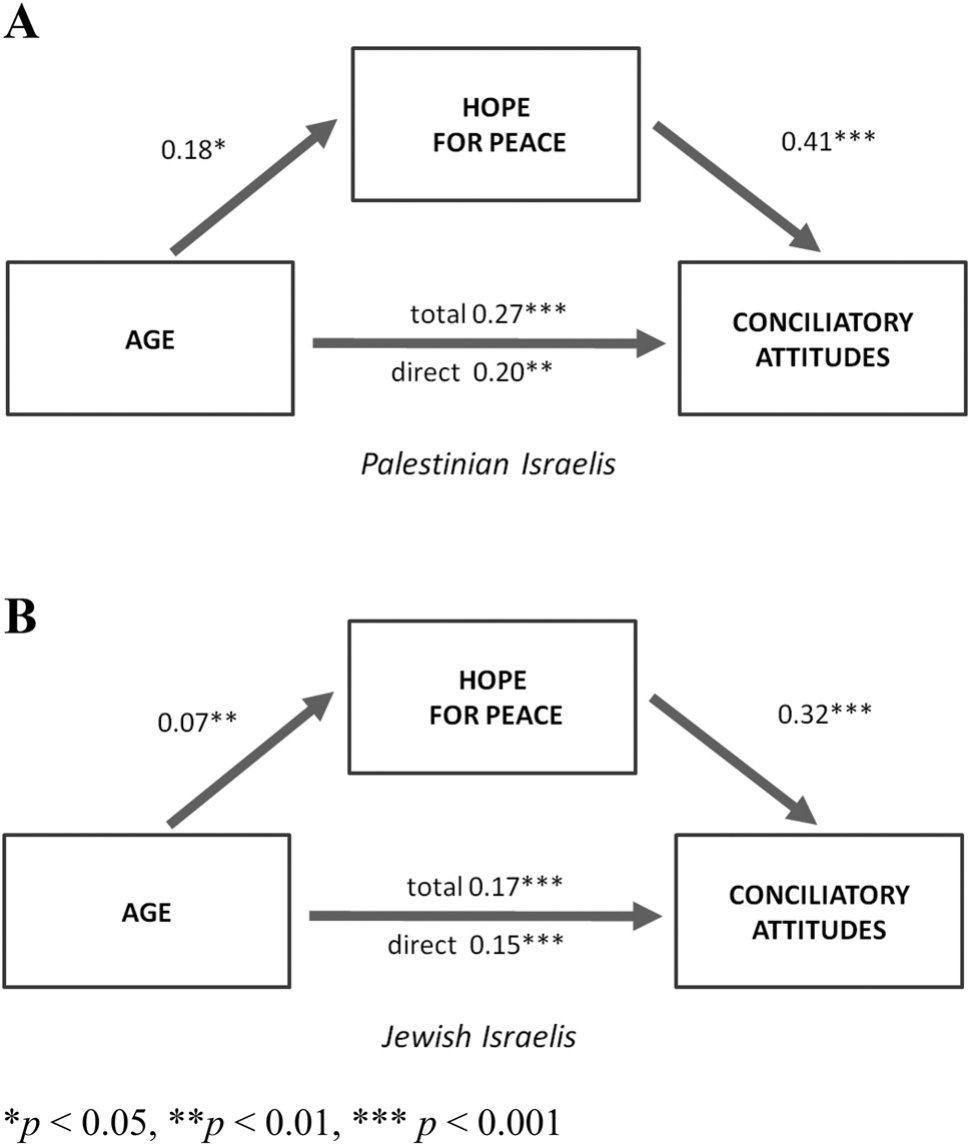
Mediation of age on conciliatory attitudes through hope for peace among Palestinian citizens of Israel (**A**) and Israeli Jews (**B**). Values are standardized beta coefficients.

As expected, older age predicted more hope for peace in both the Palestinian sample (*β* = 0.18, *SE* = 0.007, *p* = 0.02), and the Jewish sample (*β* = 0.07, *SE* = 0.003, *p* = 0.02). Next, we estimated mediation models to examine the link between age, hope for peace, and conciliatory attitudes in both samples (Fig. 2). We found that age is associated with higher levels of conciliatory attitudes, which is mediated by hope for peace in both the Palestinian sample (indirect effect: *β* = 0.07, 95% CI 0.01–0.15) and the Jewish sample (indirect effect: *β* = 0.02, 95% CI 0.005–0.04). To enhance clearity, we omitted the paths of the covariate in the model among Jewish–Israelis (Fig. 2B). The corresponding model with covariate paths can be found in the Supplementary Material (Fig. S1).

Taken together, the findings of the first three studies indicate that age is associated with emotions and attitudes that are crucial for the resolution of long-term conflicts. However, these studies have been conducted in the context of the Israeli-Palestinian conflict and so our findings might be confounded by features unique to this case. For example, the younger age groups in this population (specifically, secular Jewish–Israelis) are exposed through military service to ‘hands-on experiences’ of conflictual interactions between Jews and Palestinians. Hence, it remains unclear whether and how our key proposition—that age relates to hope for peace and resulting conciliatory attitudes—might generalize beyond this specific population and context. Can we find the same pattern in intractable conflicts that are substantially different from the Israeli-Palestinian case?

One such conflict is the decades-long dispute in Cyprus. Though both conflicts are defined as intractable^39^, the conflict between Turkish and Greek Cypriots differs from the conflict in Palestine-Israel on historical, geopolitical, cultural, religious, and social levels^40,41^. Importantly, unlike the violent conflict in Israel/Palestine, the conflict in Cyprus has not been violent for more than 25 years. (For the sake of comparison, in the last 13 years, the number of conflict-related casualties in Cyprus was zero while approximately 6000 Palestinians and 300 Israelis were killed in the same time frame.)

### Study 4

To strengthen the generalizability of our findings, Study 4 tested whether the association between age and hope for peace can also be found in the context of the conflict in Cyprus among both Greek Cypriots (*N* = 536) and Turkish Cypriots (*N* = 549), and whether it extends to conciliatory attitudes (measured by ratings of support for peace initiatives and policies). Hope for peace was measured on a five-point scale (5 = highest level), and conciliatory attitudes on a six-point scale (6 = highest level). As in Study 3b, we also controlled for political ideology; in this case we used the ‘ethos of conflict’ as an equivalent measure that taps into people’s conflict-relevant ideology^41^.

**Figure 3:**
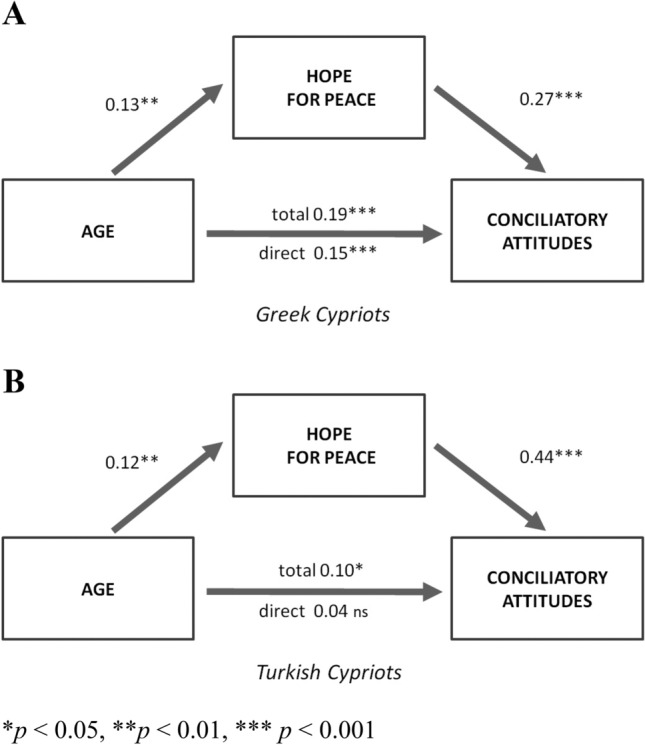
Mediation of age on conciliatory attitudes through hope for peace among Greek Cypriots (**A**) and Turkish Cypriots (**B**). Values are standardized beta coefficients.

As expected, age predicted hope for peace, such that the older one is, the more one believes in the possibility of peace. This was demonstrated among both Turkish Cypriots (*β* = 0.11, *SE* = 0.005, *p* = 0.009) and Greek Cypriots (*β* = 0.13, *SE* = 0.004, *p* = 0.005), even when controlling for Cypriots’ conflict-relevant ideology. In addition, hope for peace mediated the effect of age on conciliatory attitudes for both Greek Cypriots (Fig. 3A) and Turkish Cypriots (Fig. 3B). The association between age and conciliatory attitudes was significantly reduced when hope for peace was entered into the model among Greek Cypriots (indirect effect: *β* = 0.04, 95% CI 0.001–0.004), and completely dissapeared when hope was entered into the model in the Turkish Cypriot sample (indirect effect: *β* = 0.05, 95% CI 0.001–0.07). Models with covariate paths are provided as Supplementary Material (Figs. S2 and S3).

Study 4 replicated the results from the studies conducted in Israel-Palestine (Studies 1–3) in a different, unrelated, intractable conflict. These findings strengthen our proposition that the generational gap in hope for peace may be a general phenomenon in intractable conflicts. If hope for peace gradually increases with age, irrespective of contextual factors, is there a way to accelerate this process to overcome the challenges associated with young generations’ hopelessness?

While biological age is a given by nature, it is possible to virtually change people’s age using virtual reality technology. Virtual reality offers the unique possibility to temporarily substitute participants’ physical body with a virtual body^42^, which can have very different characteristics than their own, such as a different age. Previous research has shown that embodiment in a virtual body of a different age alters participants’ cognition and behavior in accordance with their virtual age*.* For instance, embodying adults in the virtual body of a child has been found to result in self-associations of child-like attributes and an overestimation of object sizes^43^. Another recent study has shown that embodiment in an old virtual body reduces young adults’ walking speed^44^. Building on these previous findings, we explored whether a virtual aging simulation would trigger similar effects in the context of intractable conflicts and make young people more hopeful about a peaceful resolution of the conflict in which they are involved.

### Study 5

Study 5 experimentally tested the effect of a virtual aging simulation on young people’s hope for peace in the context of the Israeli-Palestinian conflict. We used a newly developed virtual embodiment technique based on 180-degree stereoscopic video^45^ to provide young Jewish-Israelis (*N* = 69) in their early to mid-twenties with a virtual aging experience. Participants were embodied in a gender-matched photorealistic virtual body of either an 80-year-old (virtual aging condition) or a 25-year-old (control condition). Importantly, the virtual embodiment experience made no reference to the conflict whatsoever.

We tested hope for peace, general hope about the future, and optimism as dependent variables. All variables were measured on a six-point scale (6 = highest level). As the variables deviated from normal distribution, we used non-parametric tests (Mann–Whitney U-Test) for median comparison between the conditions.

Participants in the virtual aging condition (*M*_*d*_ = 3.67) showed significantly higher levels of hope for peace after the embodiment experience compared to the control group (*M*_*d*_ = 3.33), *U* = 782.5, *SE* = 82.62, *p* = 0.02. However, consistent with the findings of Study 2 for biological age, there was no significant difference between the two conditions regarding participants’ general hope about the future (*M*_*d*_ = 4.00), *U* = 658, *SE* = 76.65, *p* = 0.41, nor their optimism (*M*_*d*_ = 3.25), *U* = 516.5, *SE* = 82.17, *p* = 0.34 (The Median values are identical in both conditions).

While these findings provide initial evidence that a virtual aging experience can increase hope for peace in young people, it remains unclear how exactly it does so. A potential mechanism is that a virtual aging experience helps young people to create a clearer vision of the future. This hypothesis is based on research showing that the distant future self tends to be mentally represented in third person compared to the first-person representation of the current or near-future self^46,47^. When distant future events are simulated from a third-person vantage point, they tend to be mentally represented in an abstract and schematic way^46^. Experimentally induced shifts from third- to first-person vantage points (as created by an embodied virtual aging experience), may therefore lead to a more detailed representation of future events, which according to Construal Level Theory^48–50^ may result in a greater perceived probability of their occurrence.

Beyond these expected changes in how future events are imagined, previous research has shown that an experimentally increased connection between the current and future selves influences the decisions and behaviors in the here and now^51^. In other words, having a clearer vision of the future is likely to motivate people to act in ways that help to attain the desirable (or prevent an undesirable) anticipated future. This has been demonstrated in a previous study^52^ that showed that virtually embodying an age-progressed version of the self reduced subsequent delinquent behavior. Thus, it is possible that a virtual aging simulation may not only increase hope for a peaceful future but also motivate a shift towards more conciliatory attitudes required to achieve this goal.

### Study 6

Study 6 aimed to replicate the virtual aging effect on hope for peace, and to examine whether it extends to greater conciliatory attitudes (measured by ratings of support for peace-promoting policies). In addition, Study 6 explored the hypothesized mechanism underlying these effects. We used the same virtual embodiment technique as in Study 5 to embody Jewish-Israeli participants (*N* = 81) in their twenties either in an 80-year-old virtual body (virtual aging condition) or a virtual body of their own age (control condition). All variables except for conciliatory attitudes (Shapiro–Wilk (81) = 0.98, *p* = 0.16) deviated from normal distribution. Therefore, we used non-parametric tests (Mann–Whitney U-Test) for median comparison between the conditions. Descriptive statistics are shown in Table 1.

**Table 1:** Means, standard deviations, and median values of outcome and mediator variables in Study 6.

Variables		Virtual aging			Control		
		N	Mean (SD)	M_d_	N	Mean (SD)	M_d_
1	Conciliatory attitudes	42	3.94 (0.91)	3.95	39	3.51 (0.95)	3.27
2	Hope for peace	42	4.44 (0.95)	4.60	39	4.07 (0.82)	4.00
3	General hope	42	4.88 (1.23)	5.00	39	4.87 (1.30)	5.00
4	Optimism	42	4.32 (1.10)	4.25	39	4.65 (1.01)	5.00
5	Negativity bias (collective future)	40	35.83 (31.48)	33.33	38	37.28 (35.39)	33.33
6	Detail (positive collective future)	37	4.23 (2.35)	3.50	31	2.80 (1.07)	3.00
7	Probability (positive collect. future)	37	2.85 (0.83)	3.00	31	2.66 (0.92)	2.67

Variables 1–4 are measured on a six-point scale (6 = highest level). Level of detail (word count measure) and perceived probability (four-point scale; 4 = very likely) are based on descriptions of anticipated positive collective future events. Missing data in variable 5 are due to missing or invalid responses. Missing data in variables 6 and 7 are due to cases in which either all generated collective future events were rated as negative, or responses were missing or invalid.

Consistent with the findings of Study 5, the conditions neither significantly differed regarding general hope about the future, *U* = 818.5, *p* = 0.996, nor dispositional optimism, *U* = 652.5, *p* = 0.11, but hope for peace was significantly higher after the virtual aging experience compared to the control condition, *U* = 611.5, *p* = 0.049. Importantly, virtual aging also resulted in significantly greater conciliatory attitudes compared to the control group, *F*(1, 77) = 4.07, *p* = 0.047, *d* = 0.46.

To examine the potential underlying mechanism, we tested whether a virtual aging experience changed the way young participants perceive their collective future. This was measured using freely generated events that could occur in the collective future that were later categorized as positive or negative by two independent raters. As participants were free to imagine events that could happen at any point in time in their collective future, there was a large variance in the temporal distance of the generated events, ranging between 0 and 980 years from the present. (We used the same measurements for personal future events. The conditions did not significantly differ regarding the level of detail in which personal future events were represented nor the perceived probability of their occurrence. These results are reported in Supplementary Table S1).

Virtual aging did not lead to a generally more positive vision of the collective future compared to the control condition,* U* = 751.00, *p* = 0.93. However, the virtual aging condition resulted in significantly more detailed descriptions of anticipated *positive* collective future events than the control condition, *U* = 378.00, *p* = 0.02. In line with the predictions of Construal Level Theory^48,49^, a more detailed vision of positive collective future events was positively correlated with a greater perceived probability of their occurrence, *r* = 0.25, *p* = 0.04 (Partial Spearman correlation: *ρ* = 0.21, *p* = 0.09; consistent with the probability-as-distance framework based on Construal Level Theory^48,49,53^, the temporal distance at which the events were expected to occur was negatively correlated with the perceived probability of their occurrence, *ρ* = − 0.41, *p* = 0.001. To control for these variations, we included temporal distance as a control variable in a Partial Correlation analysis.)

Perceived probability of positive collective future events, in turn, was positively correlated with hope for peace, *ρ* = 0.26,* p* = 0.03, and dispositional optimism, *ρ* = 0.34, *p* = 0.005. No significant differences were found between the conditions for anticipated *negative* collective future events (statistics are provided in Supplementary Table S2).

**Figure 4:**
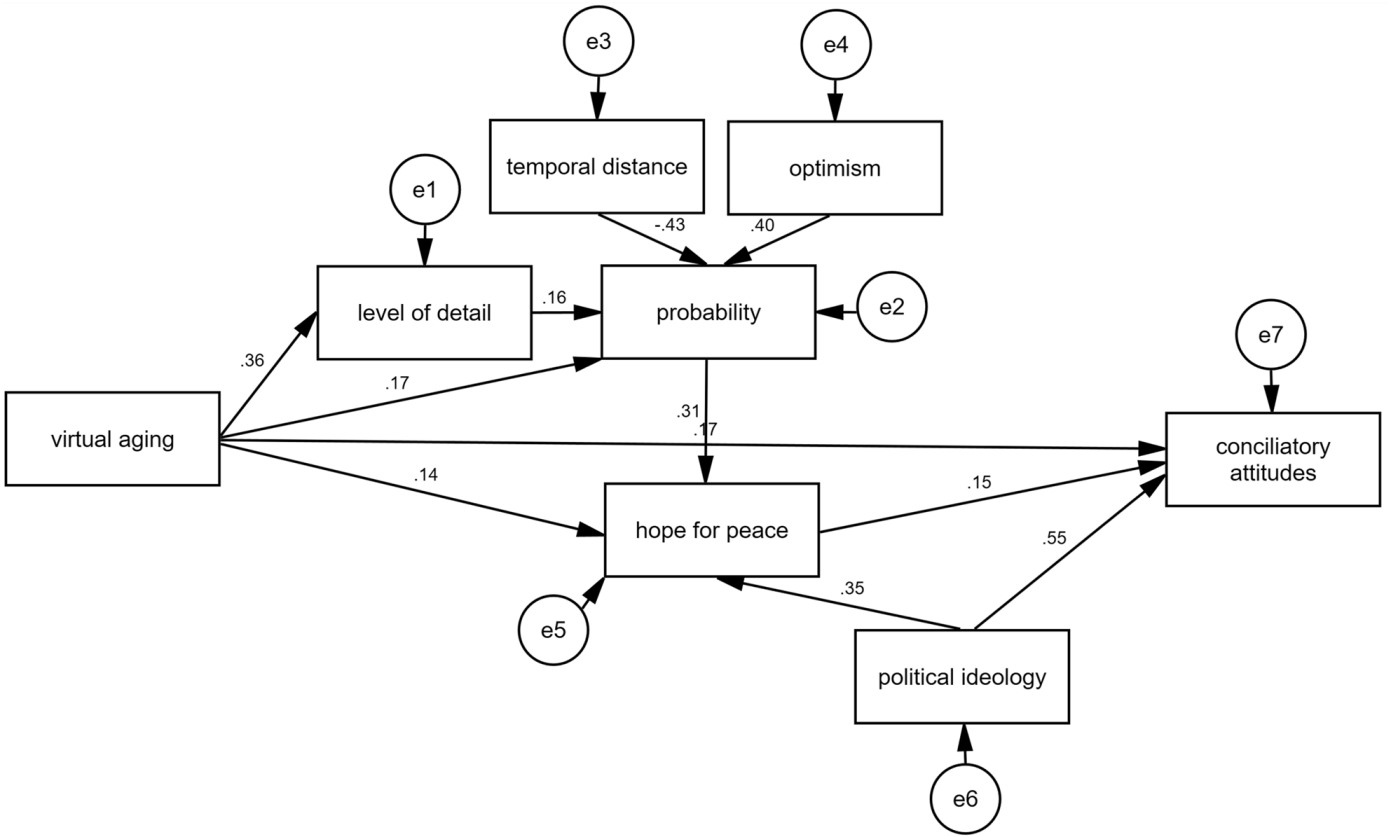
Path analysis model of the virtual aging effect on hope for peace and conciliatory attitudes. The model is based on data of anticipated positive collective future events*.* Boxes represent the variables where *virtual aging* is coded as 1 = virtual aging, 0 = control, *level of detail* is the word count of event descriptions, *probability* is the perceived probability of generated events with 1 (lowest) to 4 (highest), *temporal distance* is the time of estimated event occurrence in years from the present, *optimism* is 1 (lowest) to 6 (highest), *hope for peace* is 1 (lowest) to 6 (highest), *political ideology* is 1 (very right-wing) to 7 (very left-wing), and *conciliatory attitudes* is the level of support for peace-promoting policies with 1 (lowest) to 6 (highest). The circles represent the random error terms.

These findings provide partial evidence for the hypothesized mechanism related to changes in the representation of a positive collective future through a virtual aging experience and suggest additional variables that need to be taken into consideration. Following previous studies examining the complex underlying processes of virtual embodiment effects (e.g.,^54,55^), we tested this extended conceptual model using path analysis. (While it is theoretically possible that virtual aging makes a positive collective future seem less hypothetical and therefore more concrete, the model testing this reverse direction of causality was not statistically significant.) The path model is shown in Fig. 4. Corresponding statistics are provided in Supplementary Table S3. The model is considered a good fit: *Χ*^*2*^(17) = 14.48, *p* = 0.63; CFI = 1.00; TLI = 1.06; RMSEA < 0.001. A test of mediation of virtual aging on conciliatory attitudes through level of detail and perceived probability of positive collective future events is provided in Supplementary Table S4.

## Discussion

The reported research shows a consistent pattern of lower levels of hope for peace and resulting lower levels of conciliatory attitudes among younger (vs. older) generations in two unrelated conflicts in Israel/Palestine and Cyprus, that differ in historical, geopolitical, cultural, religious, and social contexts as well as intensity and scope. This pattern held in all four communities: Jewish–Israelis, Palestinian–Israelis, Turkish Cypriots, and Greek Cypriots. These findings indicate that the young generations’ hopelessness may be a general phenomenon in long-term conflicts as they occurred irrespective of context-dependent variables. Moreover, these generational differences appear to be persistent over time as shown in Study 1. The finding that the generational gap in hope for peace significantly widened over time is concerning and highlights the importance of instilling hope in young generations as they play a critical role in advancing peace processes.

While age was consistently associated with hope for peace, we found no significant correlation between age and dispositional optimism or general hope about the future. Optimism may be conceptualized as a personal trait that is determined by individual factors other than age as indicated by previous research that failed to find a linear relationship between age and optimism^56^. Hope, as measured in the current studies, on the other hand, may be considered as an emotional state that tends to be more dynamic. General hope about the future can encompass a wide array of future scenarios, including both anticipated personal and collective future events, and is likely to result in larger individual variations that are less affected by age. In contrast, hope for peace is defined in more specific terms, with the conflict as the object of hope and the context of failures and disappointments as the backdrop. This common reference point may reduce individual variances in the levels of hope and make age-related variables more likely to influence the sentiment associated with the conflict and its potential resolution.

While the hopelessness of young generations of societies involved in intractable conflicts appears to improve with age, we show that this pattern can be altered using a virtual reality-based aging simulation that increased young Jewish–Israelis’ hope for peace and conciliatory attitudes. Our initial findings on the potential underlying mechanism indicate that a virtual aging experience can activate two essential cognitive processes associated with hope for peace—the ability to develop a clear vision of the future^1,37^ as indicated by a more detailed representation of positive collective future events, and the belief in the likelihood to achieve the desired goal^57^ as indicated by a greater perceived probability of positive collective future events. If we consider peace as an example of a (positive) collective future event, it is plausible that an enhanced ability (triggered by the virtual aging experience) to envision a positive collective future in more detail and perceive it as more probable also increased young people's hope for peace—defined on the cognitive level as the belief that the goal (peace) can be achieved in the future^8–10^. Moreover, we show that a virtual aging experience can also motivate goal-directed planning, as indicated by an increased support for peace-promoting policies.

In line with the basic assumptions of Construal Level Theory^48–50^, the activation of these psychological processes through a virtual aging experience can be explained by a shift in the vantage point from which the collective future is imagined. The common third-person vantage point that people tend to adopt when imagining distant future events is likely to result in an abstract and schematic representation of future events^46^. In contrast, first-person embodiment of an old virtual body may increase the vividness of the future self^52^ and enable participants to imagine the future in a more concrete or detailed way^58^.

That the virtual aging simulation only influenced the level of detail and associated perceived probability of *positive* but not of *negative* collective future events is plausible because people have been found to have a general negativity bias regarding the collective future^59,60^. Therefore, a virtual aging experience may specifically help young people to develop a more detailed representation of a *positive* collective future that is not generated as easily as a negative vision of the collective future. While it is possible that a virtual aging experience also influences the representation of a negative collective future, such potential changes may have been masked by a ceiling effect. In contrast to the common negativity bias regarding the collective future, previous research has shown that people have a general tendency to imagine their personal future as positive^61^. It is possible that the positivity bias about one’s personal future remained unaffected by the virtual aging simulation, or that such potential changes may be moderated by individual variances in aging anxiety. This might explain why we only found an effect of the virtual aging simulation on the envisioned (positive) collective future, but not on the personal future.

Importantly, the virtual aging effects on hope for peace and resulting conciliatory attitudes occurred without explicit reference to the conflict, and irrespective of participants’ political ideology. Such indirect interventions cause less rejection among individuals who hold strong negative attitudes towards the opposing group in conflict. These individuals often respond negatively to interventions that directly confront them with the conflict or the opponent^62^. Moreover, since indirect interventions do not relate to a specific context, they can be adopted to other conflicts without modification. The benefit of indirect psychological interventions has also been demonstrated in previous research that has shown that merely inducing perceptions of a changing world can increase hope for peace and conciliatory attitudes regardless of participants’ age^24^.

Overall, our findings indicate that despite generational differences in hope for peace, hope is a dynamic construct that can be induced through carefully designed interventions. Interventions that provide young people with a new perspective on the conflict in which they are involved, such as activating future-oriented thought through a virtual aging simulation, may be an effective means to break the pattern that contributes to the perpetuation of long-term conflicts and pave the way towards their peaceful resolution. Virtual reality bears great potential as a novel peace-building tool that is particularly attractive for young people and has the transformative power to foster positive change in intractable conflicts. Future research may further explore the mechanisms underlying such perspective change through virtual aging simulations and examine the durability of experimentally induced hope in young people. The current findings also point to a potential benefit of inter-generational peace-building efforts that may inspire young people to take action towards peace but have thus far only received little research attention (e.g.,^63,64^).

## Methods

All methods were performed in accordance with the relevant guidelines and regulations as outlined in the policies of the Nature Portfolio journals.

### Study 1

This first exploratory study was conducted based on publicly available data of the Peace Index project (http://www.peaceindex.org/DefaultEng.aspx) by the Guttman Center for Public Opinion and Policy Research of the Israel Democracy Institute—a longitudinal research project that monitors public sentiment on the Israeli-Palestinian conflict, relations between Jews and Arabs in Israel, and political or diplomatic events. The surveys were conducted monthly since 1994 on representative samples of the Jewish Israeli population. Each of the surveys contains subjective ratings of the probability of peace in the Israeli-Palestinian conflict (see Supplementary Material) as well as participants’ age (ranging from 18 to 100 years, *M* = 44.84, *SD* = 17.46). These data allow for studying age cohort effects over time as they are repeated cross-sectional surveys (i.e., they do not include the same individuals, but the same cohorts over time). In total 250 surveys from 1994 to 2017 among the Jewish Israeli population (*N* = 117,131) were included in the analysis. The data sets after 2017 only contain an age group variable instead of a continuous age variable and have therefore not been included in the current analysis.

#### Meta-analysis

The meta-analysis was performed on the 250 correlations in the monthly surveys using fixed effects in which the mean effect size was weighted by sample size. The regression analyzes were performed using the continuous age variable and monthly means of the aggregated ‘probability of peace’ variable. A linear time variable was created for the regression analysis that takes missing months into account.

### Study 2

Participants were 209 Israeli Jews (111 men, 96 women; 2 unspecified) between the age of 14–68 years (*M* = 40.73, *SD* = 14.82). The variables considered in this study were embedded in a larger survey study conducted in March, 2018, on age-related changes in time perceptions and future-related thought, including questions that are not directly related to the current study. Data were collected online through an Israeli panel survey company (iPanel) that offers monetary compensation for participation in surveys. The study was approved by the ethics committee of the Baruch Ivcher School of Psychology of Reichman University, Israel. All participants provided their informed consent by marking a checkbox in the online survey platform, Qualtrics.

#### Measurements

*Dispositional optimism* was measured using a short version of the Personal Optimism scale^35^. Items were rated on a six-point scale with higher ratings indicating higher levels of optimism (*α* = 0.76). *General hope about the future* was measured using a single item^34^: *“I feel hopeful about the future,”* rated on a scale from 1 (definitely not) to 6 (definitely yes). *Hope for peace* was measured using a validated hope scale^24^ consisting of five items (see Supplementary Material) rated on a six-point scale, with higher values indicating higher levels of hope (*α* = 0.86). Optimism and general hope about the future were measured prior to hope for peace.

### Study 3a

Participants were 174 Palestinian citizens of Israel (80 men, 86 women; 8 unspecified) between the age of 17–86 years (*M* = 36.49, *SD* = 14.76). 75.3% defined themselves as Muslims, 13.8% as Christians, 8% indicated “other,” and 2.9% declined to answer. The variables used in the current study were embedded in a larger survey on conflict-related emotions and perceptions of historical events in the Israeli–Palestinian conflict. Data were collected in May–June, 2017, through an online survey (*n* = 123) and using printed questionnaires (*n* = 51). Participants were recruited through the researchers’ social networks using snowballing techniques and volunteered to participate in the survey study. The study was approved by the ethics committee of the Baruch Ivcher School of Psychology of Reichman University, Israel. All participants who took part in the online survey provided their informed consent by marking a checkbox in the online survey platform, Qualtrics. Participants who completed the survey on paper provided their informed consent by signing a printed form.

#### Measurements

*Hope for peace* was measured using two items (see Supplementary Material) (*α* = 0.75) that combine both cognitive (i.e., belief in the likelihood of resolution) and affective dimensions of hope (i.e., feeling of hopefulness)^6^. Consistent with previous research on the relationship between cognitive and affective dimensions of hope^6^, the two items were highly positively correlated: *r* = 0.62, *p* < 0.001. *Conciliatory attitudes* were measured using four items adapted from^65^ (see Supplementary Material) that were rated on a six-point scale, with higher valued indicating stronger support of peace-promoting policies (*α* = 0.77). The hope for peace measurement preceded the measurement of conciliatory attitudes.

#### Mediation analysis

The mediation analysis was conducted using Hayes’^66^ PROCESS macro for SPSS (version 3.0, model 4; with 5000 bootstrap iterations and 95% bias-corrected confidence intervals). Age was defined as the independent variable, conciliatory attitudes as the dependent variable, and hope for peace as the mediator.

### Study 3b

Participants were 984 Israeli Jews (507 men, 477 women) between the age of 20–66 years (*M* = 38.72, *SD* = 12.28). 51.3% self-identified as rightists, 29.9% as centrists, and 18.8% as leftists regarding their political ideology. The variables considered in the current analysis were part of a larger survey study conducted in September, 2014, including questions that are not directly related to the current study (i.e., dealing with specific issues of intergroup relations and reactions to specific conflict events). Data were collected online through an Israeli panel survey company (Midgam) that offers monetary compensation for participation in surveys.

#### Measurements

*Hope for peace* was measured using the first three items of the hope for peace scale used in Study 2 (*α* = 0.80). This three-items scale has been shown to be a valid measure of hope for peace in previous studies^25^. *Conciliatory attitudes* were measured by asking participants to indicate the extent to which they are willing to agree to peace-supporting policies, rated on a six-point scale (*α* = 0.81). The items are adapted from^25,65^ and are provided as Supplementary Material. *Political ideology* was measured on a seven-point scale from 1 (extreme left) to 7 (extreme right). The variable was reverse coded so that higher values indicate greater left-wing (dovish) political ideology. The hope for peace measurement preceded the measurement of conciliatory attitudes. Political ideology (along with demographic variables) was measured at the end to prevent potential priming effects.

#### Mediation analysis

The mediation analysis was conducted using Hayes’^66^ PROCESS macro for SPSS (version 3.0, model 4; with 5000 bootstrap iterations and 95% bias-corrected confidence intervals). Age was defined as the independent variable, conciliatory attitudes as the dependent variable, hope for peace as the mediator, and political ideology as the covariate.

### Study 4

Participants were 549 Turkish Cypriots (239 women, 310 men) between the ages of 18 and 82 years (*M* = 44.7, *SD* = 15.8) and 536 Greek Cypriots (217 women, 319 men) between the ages of 18–94 years (*M* = 52.7, *SD* = 17.2) who answered a telephone survey as part of a larger study on the Cypriot conflict. The study was conducted between July and November, 2020, under the supervision of the Center for Field Studies at the University of Cyprus. The study was approved by the Cyprus National Bioethics Committee. All participants gave their informed consent by phone.

#### Measurements

*Hope for peace* was measured using a single item taken from^67^ (see also^38^), gauging the levels of participants' expectations that peace will materialize in the future, on a six-point scale (6 indicating the highest level). *Conciliatory attitudes* were measured by asking participants to rate, on a five-point scale (5 indicating the highest level), how much they are willing to support four peace-building initiatives and policies (see Supplementary Material). *Political ideology* was measured using six items from the Ethos of Conflict (EOC) scale^41^ that required participants to mark, on a five-point scale (with higher values indicating more dovish political ideology), the extent they agreed with six statements about conflict-related issues (see Supplementary Material). The EOC is used as a proxy for conflict-related political ideology because the items are comparable across rival parties (see^68^). The scale has been validated in various conflicts, including the Kurdish conflict in Turkey^69^, the conflicts in former Yugoslavia^70^, and the Israeli-Palestinian conflict^71^. The hope for peace measurement preceded the measurement of conciliatory attitudes. Political ideology (EOC scale) along with demographic variables) was measured at the end to prevent potential priming effects.

#### Mediation analysis

Mediation analysis was conducted using STATA bootstrap command (5000 iterations and 95% bias-corrected confidence intervals). Age was entered as the independent variable, conciliatory attitudes as the dependent variable, expectations of peace as the mediator, and political ideology (EOC) as the covariate.

### Study 5

Sixty-nine students (41 women, 28 men) between the age of 21–28 years (*M* = 23.57, *SD* = 1.83) were recruited at Reichman University, Israel, to participate in the study for course credits. The study was conducted between June and December, 2017. All participants were Jewish–Israelis. 40.6% self-identified as rightists, 33.3% as centrists, and 26.1% as leftists regarding their political ideology. Participants were randomly assigned to the virtual aging condition (*n* = 35) or the control group (*n* = 34). Gender was equally distributed across the two conditions. The conditions did not significantly differ regarding participants’ age, *t*(67) = 0.42, *p* = 0.68, or political ideology, *t*(67) = 0.98, *p* = 0.33. The study was approved by the ethics committee of the Baruch Ivcher School of Psychology of Reichman University, Israel. All participants provided their informed consent by marking a checkbox in the online survey platform, Qualtrics.

#### Virtual embodiment technique

We used a novel virtual embodiment technique, developed and validated by the first author (BSH) and two co-authors (DHL and DF)^45^, using 180-degree stereoscopic video that creates a photorealistic illusion of owning a virtual body (see Movie S1 for a demonstration of the experimental procedure). When wearing a virtual reality headset, participants saw a virtual body (i.e., an actor’s body filmed using 180-degree video) from first-person perspective substituting their own physical body. They also saw the reflection of their virtual body in a mirror placed next to them, without showing the head of the virtual body. A virtual female interlocutor sitting in front of them guided them through the experience through touch and narration. While seeing the virtual hands being touched by the virtual interlocutor, the experimenter synchronously touched the participant’s physical hands to induce a multi-sensory connection with the virtual body; adapting the procedure used in the rubber hand illusion^72^ to induce a sense of ownership over an artificial body part. A narrative layer added contextual meaning to the embodiment experience of the respective virtual age. Movies S2 and S3 show the content of the embodiment experience (as seen from the participant’s point of view) in the virtual aging condition (Movie S2) and the control condition (Movie S3). Informed consent was obtained from the actress (subjects present in movies) for publication of identifying information/images in an online open-access publication. All virtual bodies matched participants’ gender. The embodiment experience in each condition was of equal duration (three minutes).

#### Measurements

*Hope for peace* was measured using the same three-item scale as used in Study 3b (*α* = 0.57). *General hope about the future*^34^ (single item) and *dispositional optimism*^35^ (*α* = 0.52) were measured using the same scales as used in Study 2. *Political ideology* was measured using the same seven-point scale as used in Study 3b. Optimism and general hope for the future were measured prior to hope for peace. Political ideology (along with demographic variables) was measured at the end to prevent potential priming effects.

### Study 6

Eighty-four Jewish Israelis participated in the study, which was conducted in December, 2020—a time marked by the COVID-19 pandemic and the signing of normalization agreements between Israel and Arab countries. Participants were recruited through the participant pool of Reichman University and through social media. All participants were paid 30 Shekels (about 9 US$) for their participation and 20 Shekels (about 6 US$) for each participant that they referred to the study. Three participants had to be removed due to technical problems. The remaining sample (*N* = 81) consisted of 63 men and 19 women between the age of 18 to 28 years (*M* = 24.14, *SD* = 2.69). 32.1% self-identified as rightists, 23.8% as centrists, and 40.5% as leftists regarding their political ideology. Participants were randomly assigned to the virtual aging (*n* = 42) or control condition (*n* = 39). All virtual bodies matched participants’ gender. The conditions did not significantly differ regarding participants’ gender, *Χ*^*2*^(1) = 1.27, *p* = 0.26, age, *t*(79) = 0.02, *p* = 0.98, or political ideology, *t*(79) = 0.49, *p* = 0.63. The study was approved by the ethics committee of the Baruch Ivcher School of Psychology at Reichman University, Israel. All participants provided their informed consent by signing a printed form.

#### Measurements

*Hope for peace* was measured using the same five-items scale (*19*) as in Study 2 (*α* = 0.74). *General hope about the future*^34^ (single-item) and *dispositional optimism*^35^ (*α* = 0.78) were measured using the same scales as in Studies 2 and 5. *Conciliatory attitudes* were measured using 11 items adapted from^73^ that were updated regarding the current situation of the Israeli-Palestinian conflict (*α* = 0.84) (see Supplementary Material). *Political ideology* was measured using the same seven-point scale as used in Studies 3b and 5.

To test the potential mechanism, participants were asked to imagine and describe three events that could happen in their collective future and three events in their personal future. We opted to let participants generate any collective future events (whether positive or negative) rather than asking specifically about the conflict-related future in order not to bias responses, and to explore a potential general mechanism underlying the virtual aging effect beyond the specific context of the conflict. A peaceful future is a possible scenario of a positive collective future, and 32.85% of positive collective future events referred to peace. For each generated event, participants estimated the year in which the event might occur (*temporal distance*) and rated the *perceived probability* of the event’s occurrence on a four-point scale (1 = very unlikely, 2 = unlikely, 3 = likely, 4 = very likely). Two independent raters marked the generated events as positive (+ 1) or negative (− 1). Interrater reliability (mean ICC = 0.88 for personal future events; mean ICC = 0.93 for collective future events) was sufficiently high to average the ratings. Events that received a mean valence rating of 0 were excluded from the analysis as they could not be clearly categorized as positive or negative. Personal events that were entered as collective events (and vice versa) as well as responses that were not a description of an event, such as ‘I don’t know’ statements were excluded from the analysis. We calculated a *negativity bias index* (i.e., the proportion of negative events) by dividing the number of negative events by the total number of valid event descriptions. As a quantitative measure of the *level of detail* of event representations, we counted the words of each event description, and averaged the word count for each type of event. We adopted a procedure used by^74^ and only included content words (adjectives, adverbs, nouns, and verbs) in the word count, while removing function words (pronouns, articles, and conjunctions) based on a standard list of Hebrew ‘stop words’ used in natural language processing (https://nlp.johnsnowlabs.com/2020/07/14/stopwords_he.html). Compound nouns, such as ‘prime minister’ or ‘Middle East’ as well as person names, such as ‘Mahmoud Abbas’ were counted as one word. The "future events" generation task including ratings of the estimated year and probability of occurrence was presented prior to the ratings of optimism, general hope about the future, and hope for peace. Conciliatory attitudes were measured afterwards. Political ideology (along with demographic variables) was measured at the end to prevent potential priming effects.

*Path analysis* was conducted using the AMOS SPSS Structural Equation Modeling software (version 25).

## Supplementary Information

Supplementary Information.

Supplementary Video S1.

Supplementary Video S2.

Supplementary Video S3.

## Data Availability

The dataset of the Peace Index Surveys analyzed in Study 1 is publicly available at https://dataisrael.idi.org.il (Search Query: "I'm looking for…": Questionnaire title; "Advanced Search": By years: Conduct between Year 1994 and Year 2017; "Subject": Peace Index). The datasets of Studies 2–6 are available from the first author on request.
