# Supplementary material for: Young generations’ hopelessness perpetuates long-term conflicts
Source: Sci Rep. 2023 Mar 25;13:4926. doi: 10.1038/s41598-023-31667-9 (PMC10039691; doi:10.1038/s41598-023-31667-9)
Supplement: Supplementary file 1 — Supplementary Information. [file 41598_2023_31667_MOESM1_ESM.docx]

**Supplementary Material**

**Young Generations' Hopelessness Perpetuates Long-term Conflicts**

Béatrice S. Hasler, Oded A. Leshem, Yossi Hasson, Daniel H. Landau, Yara Krayem, Chen Blatansky, Guy Baratz, Doron Friedman, Charis Psaltis, Huseyin Cakal, Smadar Cohen-Chen, and Eran Halperin

**This PDF file includes:**

Measurement scales

Figures S1 to S3

Tables S1 to S4

Captions for Movies S1 to S3

**Measurement scales**

Study 1

*Hope for peace* was measured as the subjectively perceived probability of peace using single items. From 1994 to 2001 the item was *“Do you believe or not believe that in coming years there will be peace between Israel and the Arabs?”* rated on a scale from 1 (certain there will be peace) to 5 (certain there will not be peace). From 2002 to 2017, the item was phrased as *“Do you believe or do you not believe that negotiations between Israel and the Palestinian Authority will lead to peace between Israel and the Palestinians in the coming years?”* rated on a scale from 1 (believe it a lot) to 4 (completely do not believe it). To create an aggregated ‘perceived probability of peace’ variable over the entire period, responses were reverse-coded and then normalized to a range of 0 (indicating low probability of peace) to 1 (indicating high probability of peace) using the formula: $z$_i_$=\frac{x_{i} -\min(x)}{\max\left( x \right)- \min(x)}$

Study 2

*Hope for peace* was measured using the following items: 1) *“I am hopeful regarding the end of the Israeli-Palestinian conflict,”* 2) *“I do not expect ever to achieve peace with the Palestinians”* (reverse coded), 3) *“There is no use in really trying to end the conflict because it probably won’t happen”* (reverse coded), 4) *“Under certain circumstances, and if all core issues are resolved, the conflict can be resolved peacefully in the future,”* and 5) *“It is clear to me that the Israeli-Palestinian conflict is hopeless”* (reverse coded). The items were rated on a scale from 1 (strongly disagree) to 6 (strongly agree).

Study 3a

*Hope for peace* was measured using two items. The first item, *“I have hope that the Israeli-Palestinian conflict can be resolved peacefully”* was rated on a six-point scale from 1 (strongly disagree) to 6 (strongly agree). The second item was a graphical slider measuring subjective ratings of the probability of peace: *“Please rate from 0 to 100 what the chances are in your opinion that the Israeli-Palestinian conflict will end peacefully in the next 20 years.”* The first item was converted into a scale from 0 to 100 in order to aggregate the two items.

*Conciliatory attitudes* were measured using the following items: 1) *“The establishment of a Palestinian state including the West Bank and Gaza, with the Palestinians of 1948 remaining citizens of the State of Israel,”* 2) *“Waiving the right of return to Israel in return for an Israeli-Palestinian peace agreement,”* and 3) *“The division of Jerusalem with an Eastern section under Palestinian rule and a Western section under Israeli rule, in return for the establishment of a Palestinian state within the 1967 borders.”* Agreement to each of these peace-supporting policies was rated on a scale from 1 (not at all) to 6 (to a very large extent).

Study 3b

*Hope for peace* was measured using the first three items of the hope for peace scale used in Study 2.

*Conciliatory attitudes* were measured using four items: 1) *“Israel should allow funds to be channeled to Palestinians in Gaza and the West Bank from peace-promoting international organizations for infrastructure development there,”* 2) *“Israel should promote joint economic and social projects for Israelis and Palestinians,”* 3) *“Israel should allow Palestinians access to roads in the West Bank,”* and 4) *“Israel should freeze settlement construction in order to advance negotiations with the Palestinians.”* Agreement to each of these peace-supporting policies was rated on a scale from 1 (strongly disagree) to 6 (strongly agree).

Study 4

*Hope for peace* was measured using a single item rated on a six-point scale (1 = very unlikely, 6 = very likely): *"Please indicate to what extent you expect that peace will be achieved (according to your definition and understanding)."*

*Conciliatory attitudes* were measured using ratings (on a five-point scale, with higher values indicating greater levels of support) of four peace-building initiatives and policies: 1) *“Compromise on land issues in order to reach peace,”* 2) *“Vote in the next elections for a candidate or party that is willing to make concessions in order to reach peace,”* 3) *“When possible, take part in a joint demonstration demanding peace,”* and 3) *“Support peacebuilding initiatives between the two communities”* (*α* = 0.81).

*Political ideology* was measured by ratings of the extent to which participants agreed with the following statements, on scale from 1 to 5 (adapted from the Ethos of Conflict (EOC) scale (Bar-Tal et al., 2012): 1) *“[Ingroup] have always aspired for peace,”* 2) *“[Ingroup] has always been subjected to disproportionate aggression from the side of the [outgroup],”* 3) *“I do not believe in the peaceful intentions of the [outgroup],”* 4) *“Untrustworthiness has always characterized the [outgroup],”* 5) *“We should not let the [outgroup] see that there are disagreements within our community regarding the resolution of the conflict,”* and 6) *“Encouraging loyalty towards the [ingroup] should be one of the education system’s most important goals”* (*α* = 0.72).

Study 5

*Hope for peace* was measured using the same three-item scale as used in Study 3b.

Study 6

*Hope for peace* was measured using the same five-items scale as in Study 2.

*Conciliatory attitudes* were measured by asking participants to indicate their level of agreement to each of the following statements on a scale from 1 (strongly disagree) to 6 (strongly agree): 1) *“To what extent do you support a permanent agreement with the Palestinians based on the Two State Solution that includes a return to the 1967 borders (withdrawal and evacuation of The West Bank) with various territorial exchanges?”* 2) *“In return for a full peace agreement, to what extent would you support Israel conceding control of the Arab neighborhoods in Jerusalem?”* 3) *“In exchange for a Palestinian willingness to move forward in negotiations, Israel should freeze construction in the settlements as a gesture.”* 4) *“As a gesture of goodwill towards Mahmoud Abbas, Israel should release a limited number of Palestinian prisoners (those with no ‘blood on their hands’).”* 5) *“In exchange for Palestinian recognition of Israel’s legitimacy as a state, to what extent would you support relinquishing Israel’s demand that the Palestinians recognize it as a Jewish state?”* 6) *“The Palestinians do not want a peaceful end to the conflict, and therefore there is no point in taking the proposals coming from their leaders seriously.”* (reverse coded) 7) *“East Jerusalem should be part of a united Jerusalem forever, and should not be negotiated.”* (reverse coded) 8) *“If no peace agreement that includes separation from the Palestinians is signed soon, Israel will lose its’ character of both Jewish and democratic.”* 9) *“Israel should promote joint economic and social projects for both sides.”* 10) *“Israel must reciprocate any peace initiative on the part of the Palestinians in order to clarify its commitment to resolving the conflict through negotiations.”* 11) *“It is important to continue trying different and many ways to reach a permanent agreement with the Palestinians even though the conflict has not ended so far.”*

**Supplementary Figures**

**
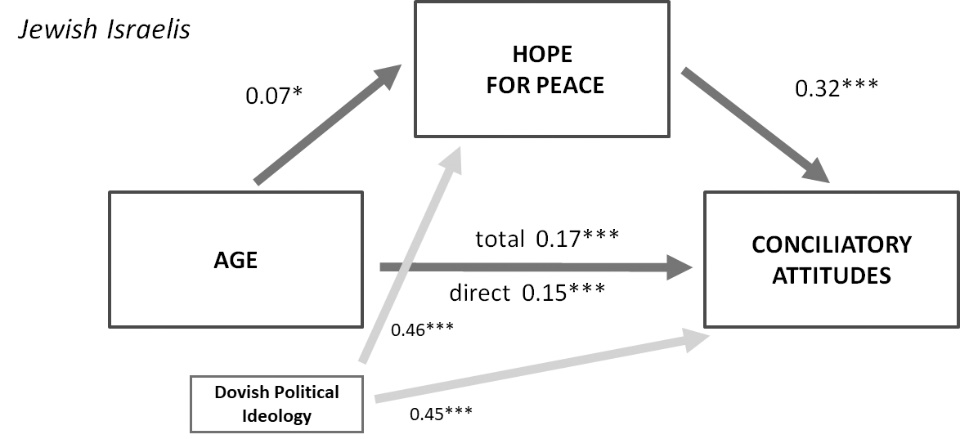
**

**Figure S1.** Mediation Model with Covariate Path (Jewish-Israeli sample; Study 3b), Corresponding to Figure 2B


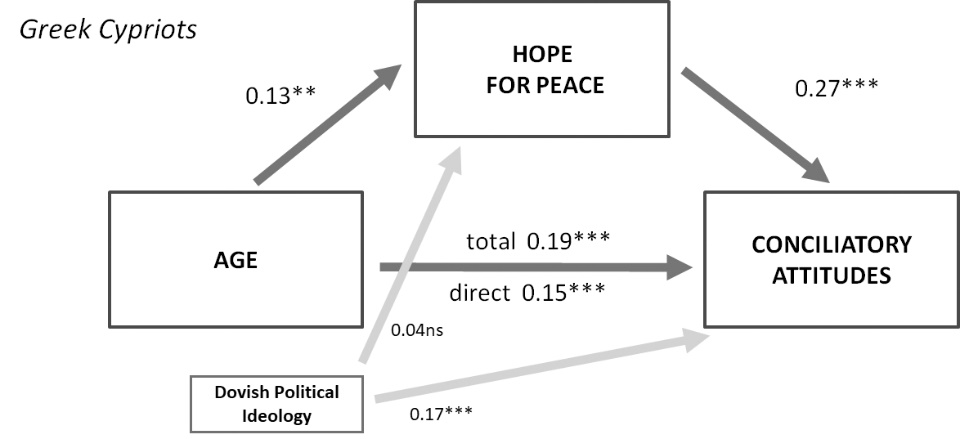


**Figure S2.** Mediation Model with Covariate Path (Greek Cypriot sample; Study 4), Corresponding to Figure 3A


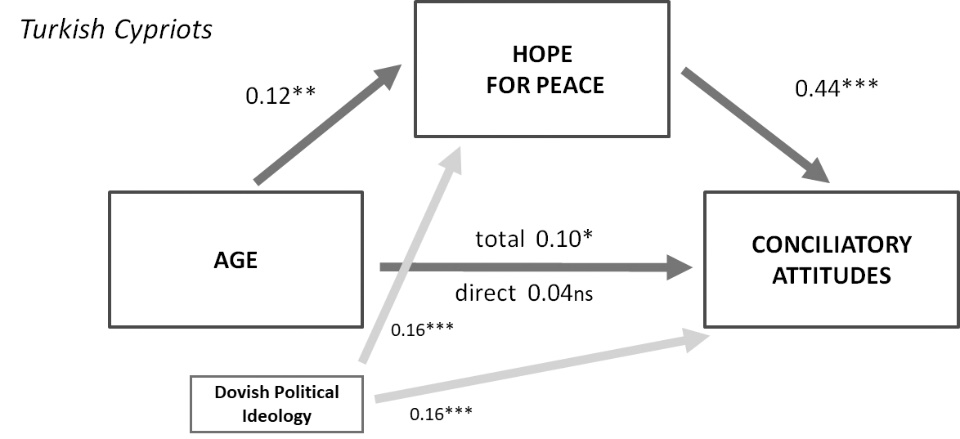


**Figure S3.** Mediation Model with Covariate Path (Turkish Cypriot sample; Study 4) Corresponding to Figure 3B

**Supplementary Tables**

**Table S1.** Means and Standard Deviations of Level of Detail, Perceived Probability and Temporal Distance (in Years from Present) of Anticipated *Personal* Future Events

|  |  | Virtual aging | | Control | |  |  |
| --- | --- | --- | --- | --- | --- | --- | --- |
| Variable | | *N* | *Mean (SD)* | *N* | *Mean (SD)* | *t* | *p* |
| *Positive personal future events* | |  |  |  |  |  |  |
|  | Level of detail | 42 | 3.54 (2.81) | 39 | 3.57 (3.35) | -0.05 | 0.96 |
|  | Perceived probability | 42 | 3.44 (0.47) | 39 | 3.47 (0.51) | -0.31 | 0.76 |
|  | Temporal distance | 42 | 7.96 (7.95) | 39 | 7.58 (9.49) | 0.63 | 0.53 |
| *Negative personal future events* | |  |  |  |  |  |  |
|  | Level of detail | 7 | 3.00 (1.63) | 6 | 3.08 (2.20) |  |  |
|  | Perceived probability | 7 | 3.57 (0.53) | 6 | 3.42 (0.49) |  |  |
|  | Temporal distance | 7 | 28.71 (22.61) | 6 | 30.50 (28.48) |  |  |

**Table S2.** Means and Standard Deviations of Level of Detail and Perceived Probability of Anticipated *Negative* Collective Future Events

|  | Virtual aging | | Control | |  |  |
| --- | --- | --- | --- | --- | --- | --- |
| *Variable* | *N* | *Mean (SD)* | *N* | *Mean (SD)* | *t* | *p* |
| Level of detail | 26 | 3.77 (3.25) | 23 | 4.36 (4.65) | -0.52 | 0.60 |
| Probability | 26 | 2.96 (0.69) | 23 | 3.03 (0.74) | -0.33 | 0.74* |

* The result remains insignificant when controlling for optimism and temporal distance, *F*(1, 43) = 0.31, *p* = 0.58.

**Table S3.** Path Model Estimates of Study 6, Corresponding to Figure 4

| *Path* | *β* | *SE* | *p* |
| --- | --- | --- | --- |
| **Level of detail** |  |  |  |
| Condition | 0.36 | 0.45 | 0.001 |
| **Probability** |  |  |  |
| Level of detail | 0.16 | 0.05 | 0.12 |
| Temporal distance | -0.43 | 0.19 | <0.001 |
| Optimism | 0.41 | 0.08 | <0.001 |
| **Hope for peace** |  |  |  |
| Probability | 0.31 | 0.11 | 0.003 |
| Political ideology | 0.35 | 0.06 | <0.001 |
| **Conciliatory attitudes** |  |  |  |
| Hope for peace | 0.15 | 0.10 | 0.10 |
| Political ideology | 0.55 | 0.06 | <0.001 |

*Note*. Estimates are standardized beta coefficients. Dependent variables are in bold, below are the respective predictors.

**Table S4.** Total, direct, and indirect effects of condition (virtual aging) on perceived probability of positive collective future events, hope for peace, and conciliatory attitudes in Study 6.

|  |  | *β* | *SE* | *LLCI, ULCI* | *p* |
| --- | --- | --- | --- | --- | --- |
| Virtual aging 🡪 Probability | |  |  |  |  |
|  | Total effect | 0.24 | 0.08 | 0.08, 0.39 | 0.01 |
|  | Direct effect | 0.19 | 0.10 | 0.01, 0.36 | 0.05 |
|  | Indirect effect | 0.06 | 0.04 | 0.002, 1.6 | 0.04 |
| Virtual aging 🡪 Hope for peace | |  |  |  |  |
|  | Total effect | 0.22 | 0.10 | 0.02, 0.41 | 0.03 |
|  | Direct effect | 0.14 | 0.10 | -0.07, 0.32 | 0.15 |
|  | Indirect effect | 0.08 | 0.04 | 0.03, 0.17 | 0.003 |
| Virtual aging 🡪 Concil. attitudes | |  |  |  |  |
|  | Total effect | 0.20 | 0.09 | 0.03, 0.38 | 0.03 |
|  | Direct effect | 0.17 | 0.09 | -0.01, 0.34 | 0.06 |
|  | Indirect effect | 0.03 | 0.03 | 0.00, 0.12 | 0.05 |

*Note.* Estimates are standardized beta coefficients, *SE* are bootstrap standard errors, LLCI and ULCI are the lower and upper bounds of 95% bias-corrected bootstrap confidence intervals. Total, direct, and indirect effects of virtual aging on perceived probability of positive collective future events, hope for peace, and willingness to compromise are estimated based on 95% bias-corrected bootstrap confidence intervals (1,000 bootstrap iterations) after performing a regression imputation on the missing data. As expected, the total effects were reduced when the mediators were added. The indirect effects of condition on probability and condition on hope for peace do not include zero, and thus indicate a significant mediation. The indirect effect of virtual aging on conciliatory attitudes has a lower-level CI of zero and is therefore only marginally significant (*p* = 0.05).

**Movie Captions**

** Movies are available at:* <https://www.dropbox.com/sh/p8jlbidhgabi5t8/AADKiTNnBG95M-I7F6xHkaZMa?dl=0>

**Movie S1.**

Demonstration of the experimental procedure used in Studies 5 and 6.

**Movie S2.**

Demonstration of the virtual aging experience used in Studies 5 and 6 from the viewpoint of the participant.

**Movie S3.**

Demonstration of the virtual embodiment experience of the control group in Studies 5 and 6 from the viewpoint of the participant.
